# The Geographical Distribution of Cutaneous Leishmaniasis Causative Agents in Iran and Its Neighboring Countries, A Review

**DOI:** 10.3389/fpubh.2020.00011

**Published:** 2020-02-18

**Authors:** Mohammad Amin Ghatee, Walter R. Taylor, Mehdi Karamian

**Affiliations:** ^1^Cellular and Molecular Research Center, Yasuj University of Medical Sciences, Yasuj, Iran; ^2^Department of Parasitology, School of Medicine, Yasuj University of Medical Sciences, Yasuj, Iran; ^3^Clinical Therapeutics Unit, Mahidol Oxford Tropical Medicine Research Unit, Bangkok, Thailand; ^4^Centre for Tropical Medicine and Global Health, Nuffield Department of Medicine, University of Oxford, Oxford, United Kingdom; ^5^Infectious Diseases Research Center, Birjand University of Medical Sciences, Birjand, Iran

**Keywords:** *Leishmania tropica*, *Leishmania major*, Iran, Turkey, Iraq, Afghanistan, Saudi Arabia, Syria

## Abstract

*Leishmania tropica* and *Leishmania major* are both the main cause of anthroponotic (ACL) and zoonotic cutaneous leishmaniasis (ZCL), respectively, in the Old World. *Leishmania infantum* and *Leishmania donovani*, which are important causes of visceral leishmaniasis, have also occasionally been reported in CL patients. The present study investigates the current distribution of causative species of CL in Iran and neighboring countries in the Middle East. There has been expansion of *L. tropica* into new urban and rural foci in Iran, with well-documented cases of visceralization, a substantial increase of CL in Syria, and the emergence of new foci and outbreaks in Turkey and Iraq, especially due to *L. major*. Civil war in Syria and Iraq, population movement, poverty, and climatic change play important roles in the changing CL distribution in this region. Control programs should adopt a multidisciplinary approach based on active surveillance and case finding, especially in vulnerable refugee populations, determination of hazard maps for CL hot points using GIS and other advanced technology, the free distribution of drugs, rodent control, and greater community engagement in poor and marginalized populations. Comprehensive molecular studies that could show the species and strains of *Leishmania* in different areas of each country can give a better view from the distribution of CL in this region.

## Introduction

Leishmaniasis is the complex disease with a spectrum of clinical manifestations, including cutaneous leishmaniasis (CL), mucocutaneous leishmaniasis (MCL), diffuse cutaneous leishmaniasis (DCL), and visceral leishmaniasis (VL, also known as kala-azar). It is transmitted by female nocturnally biting sand flies, *Phlebotomus* in the “Old World (OW)” and *Lutzomia* in the “New World (NW).” CL is more prevalent than VL and is distributed in more than 90 countries with the majority of cases occurring in South and Central America, the Mediterranean Basin and extending across the Middle East to Central Asia. The least burdensome regions include South Asia and Sub-Saharan Africa (SSA), but within SSA, East Africa (e.g., Ethiopia, Kenya) is endemic for CL and VL. VL is mostly distributed in South Asia, SSA, and South and Central America. High-burden countries, India, Bangladesh, Sudan, Ethiopia, and Brazil, account for 90% of VL patients while lower rates of VL occur in Southern Europe, Central Asia, China, and the Middle East including Iran (1).

Leishmaniasis is mainly zoonotic, but *Leishmania donovani* and *Leishmania tropica*, the causes of OWVL and OWCL, respectively, are transmitted from person to person and are also described as anthroponotic (A) VL and CL (2). The NWCL species such as *Leishmania braziliensis, Leishmania guyanensis, Leishmania amazonensis*, and *Leishmania mexicana* are zoonotic species and are endemic in Central and South America; their zoonotic reservoirs include forest rodents and sloths (3). The zoonotic reservoirs for zoonotic OWCL (ZCL) due to *Leishmania major* and *Leishmania aethiopica* are different rodent species (4) and hyraxes (2), respectively. Although humans are the main reservoir hosts of *L. tropica*, latterly it has been found in several animal species such as dogs (5), hyraxes (6), jackals, and foxes (7) and raise the question of a zoonotic cycle in some geographic regions of the Middle East.

The two principal OWCL species overlap in their geographical distribution. *L. major* extends from West Africa to Central Asia and *L. tropica* is common in the Eastern Mediterranean, Middle East, North India, Afghanistan, and northeast and South Africa (2). The clinical manifestations of OWCL vary by species. *L. aethiopica* is the main cause of DCL, which is characterized by chronicity and a poor response to conventional leishmaniasis treatments, like the antimonials but pentamdine appears more promising (8). Although in most cases the cutaneous lesions are self-healing, recovery time may last 2 to 5 years (2). DCL occurs mostly on the face (8) but oronasal mucocutaneous disease has also been documented (9).

In *L. major* infections, the ulcero-crusted form was the most frequent clinical form and occurs mainly on the lower extremities (10). The ulcers usually necrotize rapidly and multiple wet sores and severe inflammation are common and are slow to heal. Self-healing occurs in more than half of the patients within 2–8 months (2). *L. major* may also manifest as for mucocutaneous, lupoid, and sporotricoid forms.

*L. tropica* is common in the Eastern Mediterranean, Middle East, North India, Afghanistan, and northeast and South Africa (11). In patients infected by *L. tropica*, dry papular or dry ulcerative types are often observed (12) usually on the face and other exposed areas. A superficial spreading form, called leishmaniasis recidivans (LR), which presents as new lesions around an old *L. tropica* scar, and the plaque form lupoid leishmaniasis are predominantly caused by *L. tropica* (13, 14). *L. tropica* responds well to the antimonials and miltefosine (based on small case series) but LR is less responsive (13, 15). Without specific treatment, self-healing is generally longer compared to *L. major* and may last up to 1 year or more (2). Several cases of kala-azar by *L. tropica* have been reported from several countries, including India and Iran (16, 17), illustrating its propensity for invasive pathogenicity.

In recent years, there has been a substantial change in the distribution of *L. tropica* and *L. major* in Iran and its neighbors, which has challenged the prevention, control, and treatment of CL in this region. Climate change, notably drought in Iran and neighboring countries, the civil wars in Syria and Iraq, and the unstable political situation in Afghanistan have all contributed to the changes in the distribution of the disease in this endemic area of CL. Accordingly, we decided to review in detail the situation of OWCL in our region, which is one of the most important leishmaniasis foci in the world.

## The Geographical Characteristics of CL in Iran

Iran, a country of more than 80 million people, lies in the heart of the Middle East, bordered by Azerbaijan, Armenia, Turkmenistan, and the Caspian Sea in the north; Afghanistan and Pakistan in the east; the Persian Gulf and the Oman Sea in the south; Iraq in the west; and Turkey in the northwest. Northern and western Iran are covered by the Zagros and Alborz mountain ranges, respectively, and the rest of the country is covered by forests, plains, and large deserts.

VL and CL are endemic in Iran. VL is endemic in certain regions of the country, notably the northwest and southwest and several small confined regions in central, western, and southeast Iran (18–20). A new endemic focus has been reported recently in the northeast (21). VL is caused mostly by *L. infantum* and a small number of cases have been due to *L. tropica* (22).

CL is widely distributed in Iran and has been reported in 25 of 31 Iran provinces in central, west, southwest, south, southeast, east, and northeast regions (23–29).

There are two forms of CL in Iran, ZCL caused by *L. major* and ACL caused by *L. tropica*. The main animal reservoirs for *L. major* infection are desert rodents, including *Rhombomys opimus* (great gerbil), *Tatera indica* (Indian gerbil or antelope rat), *Meriones libycus* (libyan jird)*, M. persicus* (Persian jird), *M. hurrianae* (Indian desert jird), and *Nesokia indica* (short-tailed bandicoot) (4, 30, 31).

Therefore, *L. major* infection is mostly endemic in rural and arid areas and is transmitted mostly by *Phlebotomus papatasi*; however, urban foci in northeast, central, southeast, and southwest Iran are well documented as well as the reporting of sporadic cases.

By contrast, *L. tropica* is transmitted between humans and no animal reservoir host has been confirmed in Iran; however, it has been isolated rarely from dogs in this country (32). *Phlebotomus sergenti* sandfly is the main *L. tropica* vector in Iran and throughout the Middle East (33), and the ideal breeding conditions include semi-arid (annual precipitation: 226 mm) and more temperate (annual mean temperature 16.4°C) regions (34). ACL is endemic in the larger Iranian cities, including Kerman, Shiraz, Isfahan, Tehran, Mashhad, and some smaller cities such as Bam.

Data suggest overall that OWCL incidence rates have been decreasing in recent years from 37/100,000 in 2007 to 27/100,000 in 2010 and 22/100,000 in 2013 but, simultaneously, it seems that the distribution of *L. tropica* has expanded to new foci (29, 35).

The epidemiology and geographical distribution of CL in different parts of Iran are presented below.

## Distribution of CL and Related Causative Agents in Iran (Figure 1)

### South–Southeastern Iran

The highest annual occurrence of CL in Kerman province in south–southeast Iran is reported from well-known ACL foci in Bam and Kerman counties, with 1787 and 369 cases in 2008–2009, respectively, followed by Jiroft (*n* = 136), Baft (*n* = 67), Sirjan (*n* = 41), Shahre-Babak (*n* = 38), Anbarabad (*n* = 27), and Roodbare-Jonoob (24). Sharifi et al. (36) conducted a cross-sectional survey in Kerman province, using PCR to identify the *Leishmania* species causing CL; all samples from Bam, Kerman, Jiroft, Shahre-Babak, Anbarabad, and Roodbare-Jonoob were identified as *L. tropica*, and only nine (4.4%) cases of *L. major* were detected in Baft and Sirjan districts. Phylogenetic studies showed a high degree of homogeneity among *L. tropica* strains in southeast Iran (37). Hormozgan, other southeast Iranian province, has been shown to be a major foci of *L. major*; e.g., a subsample of 18 of 40 ulcers were all found to be identified as *L. major* in a study in Jask county (38).

**Figure 1:**
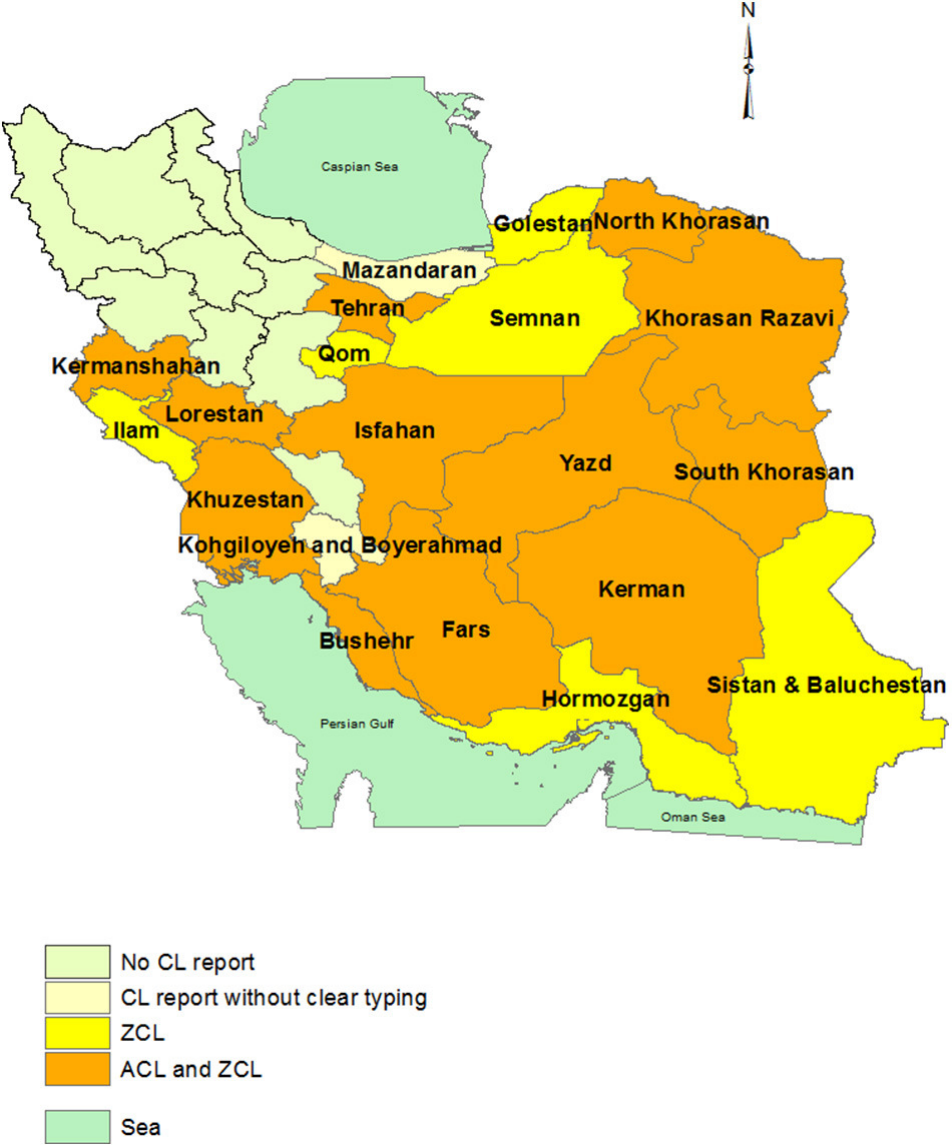
Province-based distribution of ACL and ZCL in Iran. ACL is confined to one or some counties in each province that was not shown here. The maps were generated based on the shape file layers of provinces of Iran and neighboring countries obtained from the DIVA-GIS website (http://www.diva-gis.org/gdata) by using ArcGIS version 10.1 (http://www.esri.com/arcgis).

### Southwest

Shiraz, the capital of Fars province, is an old established city and was the capital of the Zandieh Kingdom from 1751 to 1794. The city has long been recognized as endemic for ACL but no DNA-based molecular data have characterized the species until 2016. In one study, ACL was detected in the old and central parts of the city while *L. major* was reported in suburbs (27). Bushehr province is a known focus of *L. major* causing ZCL in southwest Iran (39), but a small study in Bushehr, the provincial capital, identified *L. tropica* in 6/8 CL patients (40). There are no data on the *Leishmania* species in the small province of Kohgiloyeh and Boyerahmad but a geospatial information systems (GIS)-based modeling study suggests a ZCL cycle in this part of southwest Iran (41).

### Central Iran

Yazd city, the provincial capital of Yazd province, is in close proximity to vast deserts of “Kavir-e-Loot,” and is highly endemic for ZCL and has a low burden of ACL; one study reported an ACL rate of 0.2% in patients aged 6–16 years (42). In addition to Yazd city, Ardakan and Bafgh counties are also predominantly ZCL (24, 43). Yazd province was predicted as a potential hot spot for CL by spatial modeling (44).

Isfahan city in Isfahan province is another Old Iranian city and was the capital from 1598 to 1722 during the Safavieh kingdom. The “Zayandeh rood” river that originates from the western Zagros mountain range passes through the city and irrigates the gardens and fields in and around the city. Although Isfahan is known as a city for ACL (45), *L. major* has been found in the suburbs (28).

Several rural districts of Isfahan province are known to be the most important foci of ZCL in Iran. In 2007/2008, a new ZCL focus was found in the Aran-o-Bidgol region; *L. major* was detected in 10/14 cases and *L. tropica* was detected in the remaining 4. *R. opimus* was the reservoir and *P. papatasi* was identified as the vector (46).

Tehran, the current capital of Iran, has been known traditionally as an important ACL focus (47), but we could not find any molecular studies investigating the *Leishmania* species in CL patients. Recently, ZCL cases were detected in Pakdasht county in Tehran province (27) and *L. major* has been reported from Semnan and Qom provinces in central Iran (23, 48).

### Eastern Iran

Karamian et al. (49) reported an emerging focus of ACL in Birjand county in South Khorasan province where *L. tropica* was identified in 89% (*n* = 51/57) of urban cases and 91% (*n* = 21/23) of ACL patients were rural dwellers. *L. major* was also detected in both urban and rural regions. In another study in 2016, ACL was also dominant (*n* = 55/60) in this county. In eastern Iran, *L. tropica* showed high genetic similarity with southeastern strains and may have originated from the southeast region (28).

### Western Iran

Khuzestan province is endemic for ZCL and had an overall prevalence rate of 37.39/100,000. Recently, *L. tropica* has been detected in a minority of patients with prevalence rates ranging from 3 to 10%, including in the ancient city of Shush (10% rate) in northern Khuzestan (50, 51). Data in 2013 from the mountainous province of Lorestan showed a predominance of *L. tropica*; overall, 72% (45/64) of local CL patients were infected by *L. tropica*, notably from Poldokhtar (37/43, 86%) and Kouhdasht (4/5, 80%). In Noorabad (Delfan), only 1 and 9 of 10 patients were infected with *L. tropica* and *L. major*, respectively (52).

Kermanshah province, which borders Iraq to the east, had an overall CL prevalence rate of 7.4/100,000, with most cases (47%) coming from Ghasr-e-Shirin county (on the Iran Iraq border) where the prevalence was 264.5/100,000 (53). Most (84–89%) CL cases in Kermanshah province are caused by *L. major* (54, 55). Small studies have also found *L. tropica* in Kermanshah city and Sarpole-Zahab (2/6 isolates), while seven and five isolates from Ghasr-e-Shirin and Islamabad-e-Gharb, respectively, were *L. major* (54).

Ilam province, sandwiched between Kermanshah and Khuzestan provinces and Iraq, is also endemic for *L. major*. Two studies in Ilam and Mehran counties showed that all CL cases (*n* = 61/61 and *n* = 92/92, respectively) were infected by *L. major* (56, 57).

### Northern Iran

One study in 2010 reported 62 CL cases from different health centers in Mazandaran province but did not report the species (58). However, *L. major* was detected from most (98.4%, 23/125) CL patients in the Turkman sahra region in Golestan province (prevalence rate of 32.90/100,000 in 2013), which is a well-known focus for CL (29, 59).

### Northeastern Iran

Khorasn-Razavi province is an area of hyper-endemic CL that had a prevalence rate of 64/100,000 in 2013 (30). Mashhad city, the capital of the province, is a long-standing ACL endemic focus and a recent study reconfirmed the predominance (10:1) of ACL over ZCL in 94 patients (60). In another study of 164 CL cases from Torghabe-Shandiz, southwest of Mashhad city, all patients were infected by *L. tropica* (61). Also, 10/12, 20/23, 38/49, and 53/60 CL patients from Bardaskan, Gonabad, Kashmar, and Torbat-e-Heydarieh cities and counties, respectively, which are located in the central and southern regions of this province, were infected with *L. tropica* (62, 63).

In Sarakhs, a city in the center of Sarakhs county in the northeastern Khorasan Razavi, 12 of 62 CL patients were infected by *L. tropica* and 5/12 patients had not traveled to ACL endemic areas, suggesting local transmission of ACL (64).

North Khorasan has been endemic for ZCL for decades with a prevalence rate of 50/100,000 (65). *L. tropica* was only detected in 5% (2/43) of sampled patients in this province and 95% of cases were found as ZCL (66).

Phylogenetic studies showed that northeastern *L. tropica* strains were genetically distinct from southeast–east Iranian strains but had high genetic similarity with some strains of southwest Iran (28).

## *L. tropica* and VL

Although *L. tropica* is a well-recognized cause of CL, it has rarely been associated with VL. Since the first reported case from Kenya (67), additional reports have emerged in American soldiers deployed in Iraq (68), and patients from Iran and India (16, 69, 70). Moreover, canine VL due to *L. tropica* has also been described in Iran and Morocco (32, 71).

In Iran, viscerotropic disease was first reported in 2006 in a patient from southwestern Iran, and more cases have since been reported from several regions in Iran (23, 69). The frequencies of *L. tropica*-associated VL were 1.4% in 1993–1994 (69); 9% of bone marrow samples in patients were from southeast provinces (72) and 14% of patients were from southwest Iran (17).

## Distribution of CL and Related Causative Agents in the Neighboring Countries

### Turkey

Turkey, which borders northwestern Iran, is one of the main foci of *L. tropica* in the Middle East. Aside from *L. tropica, L. infantum* causes CL in some foci (73), while *L. major* accounts for few CL cases (74).

**Figure 2:**
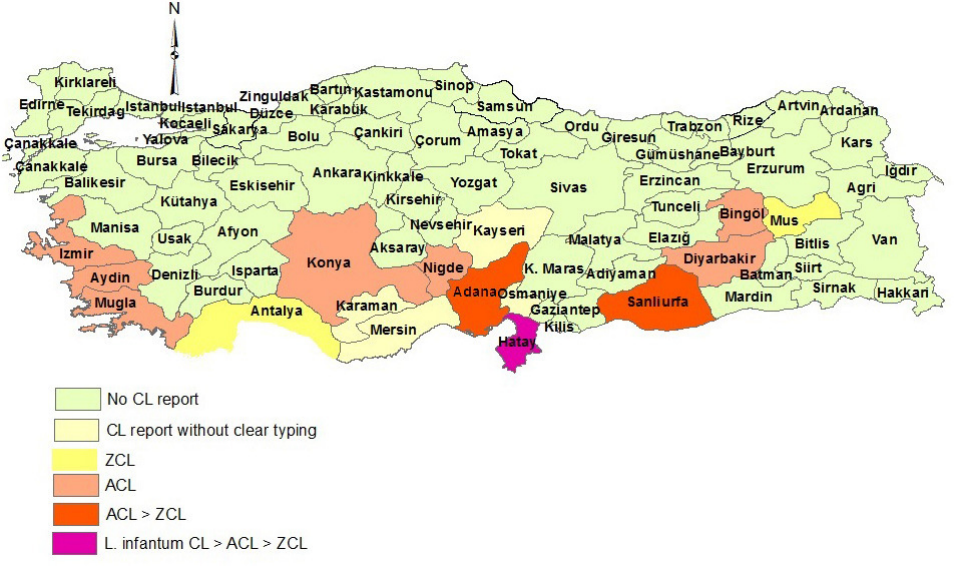
Distribution of CL types in Turkey.

The vast majority of CL patients (99%) are reported from the southeastern Anatolia region (SE-AR) and the southern Mediterranean region (MR) (75). Sanliurfa province in SE-AR borders Syria and is a highly endemic area and responsible for more than 50% of the CL burden in Turkey (Figure 2). Other CL-affected provinces include Diarbakir (SE-AR), and several MR provinces, Adana, Osmaniye, Hatay, Diarbakir, and Mersin/Icel. Low CL endemic provinces include Kahramanmaras, Antalya, Aydin, Kayseri, Nigde, and Mus provinces (73).

Based on the molecular species identification, *L. tropica* was reported from Sanliurfa and Diarbakir (southeast); Nigde (MR, south); Izmir, Aydin, and Mugla (ER, southwest); Bingol (east); Konya (Central Anatolia region, south-central); and Adana and Hatay (south), while *L. infantum* was known as the main species in Hatay province and reported from one patient in Osmaniye (south) (76–81). *L. major* has also been reported in small numbers in Mus, Hatay, Adana, Sanlirufa, and Antalya (74, 82, 83). The presence of *L. major* in southern Turkey is due to the arrival of Syrian refugees since 2011 to these areas where *L. major* has not been identified previously (83).

There is high *L. tropica* heterogeneity in Turkey with distinct clades (84–86). There are genetic similarities between northwest Iranian *L. infantum* strains and hybrid strains of *L. infantum*/*L. donovani* in Cukurova in southeast Turkey (17). The recent arrival of refugees from Syria will add additional genetic heterogeneity to the *L. tropica* genetic pool in Turkey (86). Aside from humans, *L. tropica, L. major*, and *L. infantum* have been isolated frequently in cats in Turkey and may be influenced by different ecological cycles (87, 88).

### Saudi Arabia

Saudi Arabia is another important focus of CL (89), with most cases occurring in southwest, central, and eastern parts of the country. *L. tropica* is mainly endemic in Aseer province in the southwest (90) while *L. major* is reported from Hail, Al-Hasa, Eastern, and the Riyadh provinces (91, 92).

**Figure 3:**
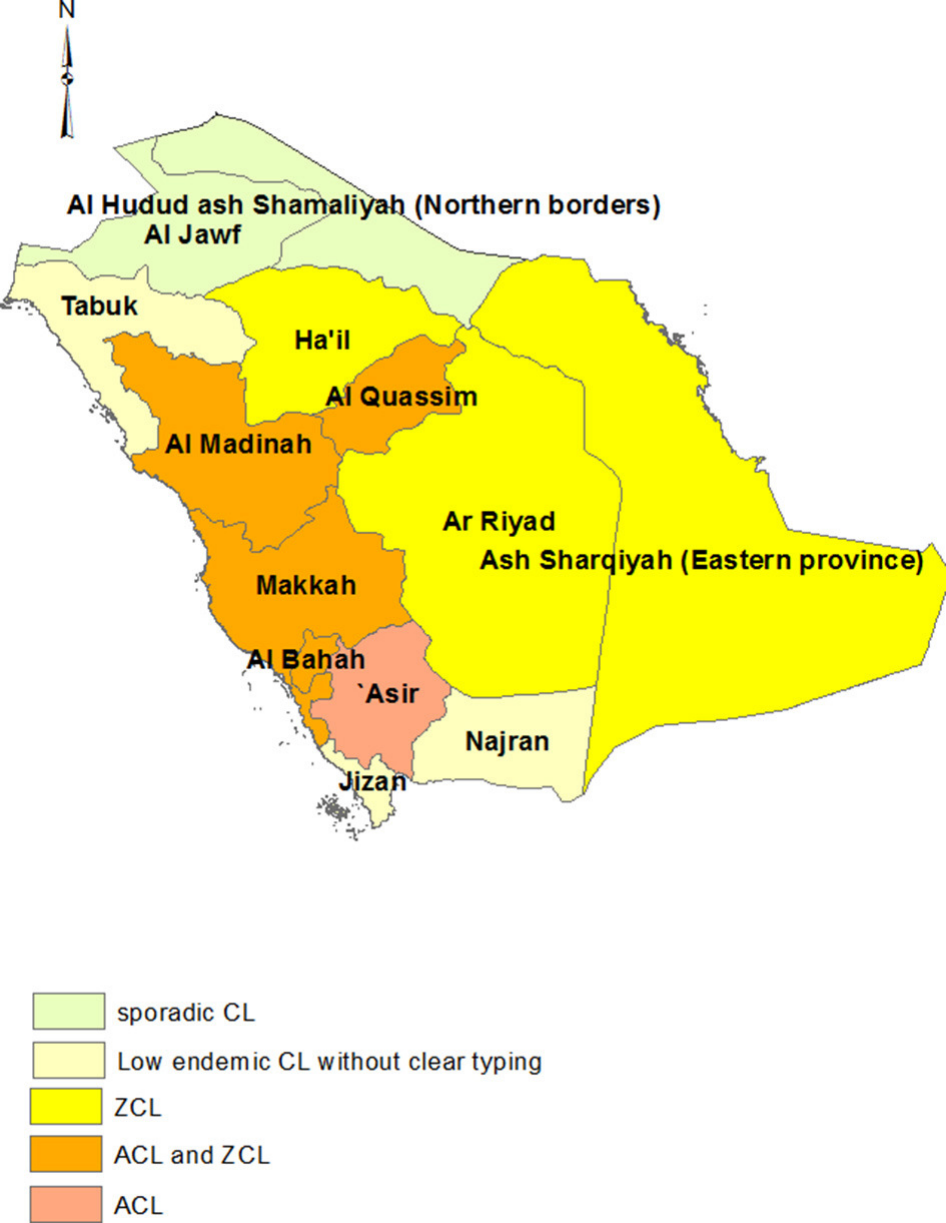
Distribution of CL types in Saudi Arabia.

Areas of mixed ACL and ZCL occur in Al-Madinah (91) and Al-Baha (93) provinces, the Taif region of Makkah provinces (94) in the southwest and Al-Qasim province in central Saudi (93). CL is declining and reported at low frequencies (mean 1.6–16.6 cases annually from 2006 to 2012) or sporadically in Tabooq, Jazan, Najran Al-Jouf, and northern borders (Figure 3) (95).

Each year, tens of thousands of pilgrims from Iran and other CL endemic countries travel to Al Madinah and Al Makkah for the holy Haj pilgrimage and usually stay for 12 to 30 days. This population may become infected with Saudi strains of *L. tropica* and *L. major* and also contribute new strains of CL to the local population. One study reported that the internal transcriber spacer (ITS) gene of *L. major* isolates from Al Madinah were 100% concordant with some Iranian *L. major* strains (96).

### Iraq

**Figure 4:**
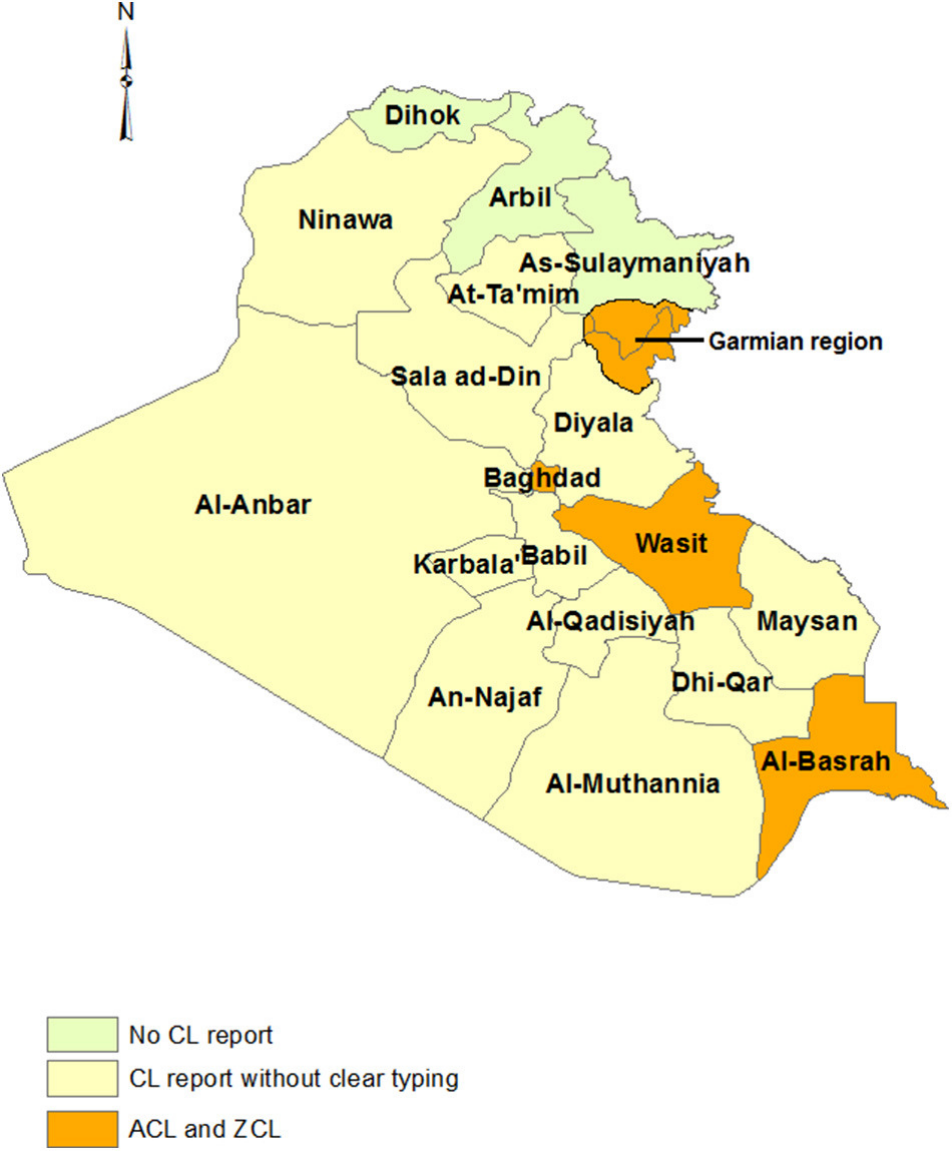
Distribution of CL types in Iraq.

Iraq borders Iran, Turkey, and Saudi Arabia. CL has been prevalent in all parts of country except in the three northeastern provinces of Sulaymaniyah, Dahuk, and Arbil (97). *L. tropica* is prevalent in the suburbs of major cities such as Baghdad and Mosul and *L. major* is reported from different regions throughout the country, although no clear species typing has been reported for most regions (Figure 4) (98). The Wasit governorate is one of the most important CL endemic areas in Iraq where the *L. major*–*L. tropica* ratio is 3:2 (99, 100). Wasit borders the western Iranian provinces of Ilam, Kermanshah, Khuzestan, and Lorestan, which harbor mostly *L. major*, and is on a main route to Karbala province, where millions of Iranian pilgrims travel annually. We could not find any phylogenetic studies of Iraqi *L. tropica* but it is likely that there are shared genotypes between eastern Iraq and western Iran. A genetic study of CL in Iraq identified *L. major* as the cause of a CL outbreak in the Garmian region in Sulaymaniyeh province from where there are no previous cases of CL (101). Garmian is close to Ghasr-e-Shirin county in Kermanshah province, Iran, a known focus of *L. major* (54). Moreover, the phylogenetic analysis of the Iraqi *L. major* showed a close genetic relationship of *L. major* from Ghasr-e-Shirin (101).

### Pakistan

**Figure 5:**
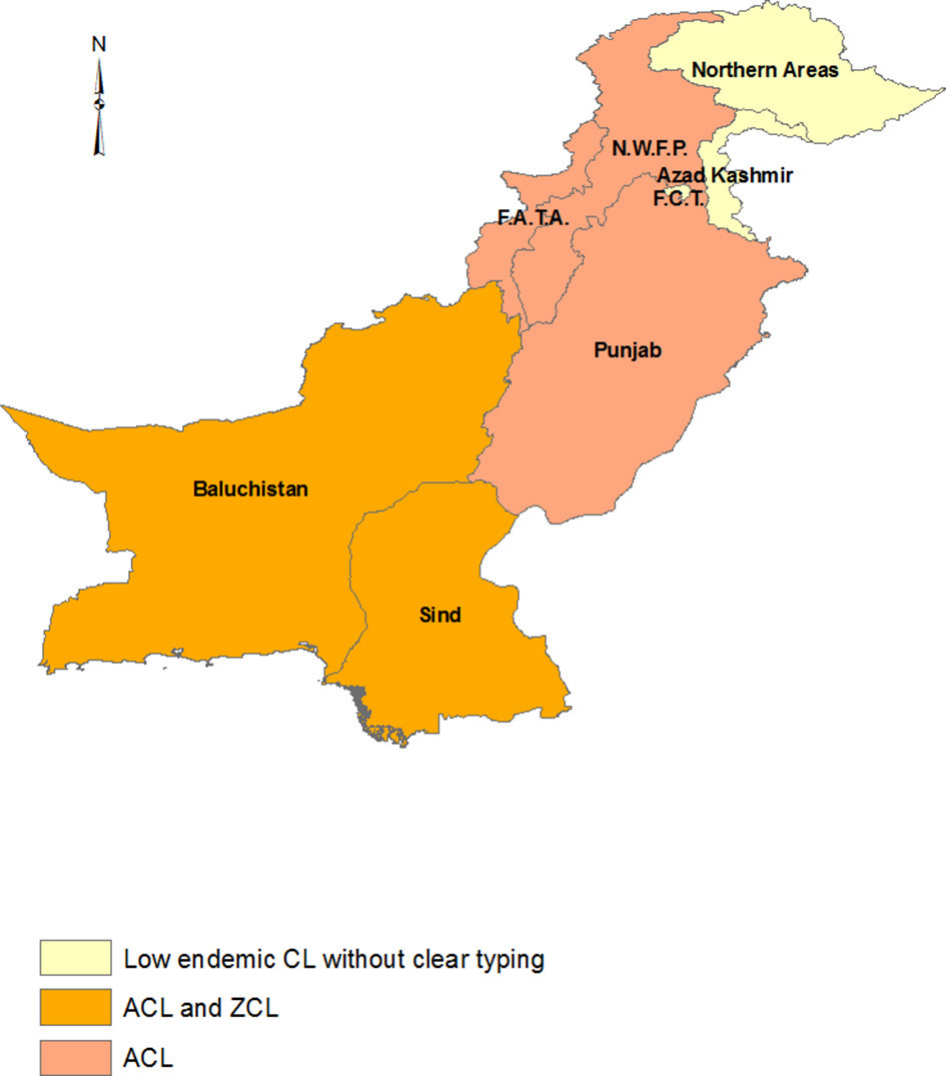
Distribution of CL types in Pakistan. The Khyber Pakhtunkhwa province was assumed the areas under N.W.F.P and F.A.T.A regions.

Pakistan, the southeast neighbor of Iran, is endemic for both ACL and ZCL. *L. tropica* is widely distributed in different parts of all provinces in this country, but notable areas of high endemicity include the Afghan refugee camps in northwest Pakistan (102), the west–northwest province of Khyber Pakhtunkhwa province (5.17%), which borders Afghanistan, and Baluchistan province, the south-SW province bordering Iran (103). *L. tropica* is also found in Punjab and Sind provinces. The main focus of *L. major* is Balochistan (104) and central parts of Sind province (Figure 5) (105) where it is mostly a disease in the plains whereas *L. tropica* is prevalent in mountainous regions and the cities (104, 106).

One genetic study revealed low genetic polymorphism for *L. tropica* from Balochistan and Sind (104). West Afghanistan (Herat province) and east (South Khorasan) and southeast (Kerman province) Iran are the main *L. tropica* foci in close proximity to Pakistan and showed higher homogeneity alongside central Iran isolates in comparison to other Iran's *L. tropica* foci (11, 28). There has not been a direct genetic comparison between Pakistan and Indian *L. tropica*, but interestingly, there is genetic similarity between southeast Iranian and Indian *L. tropica* strains (107), which raises the possibility that there may be a Pakistan–India genetic link.

### Afghanistan

Afghanistan, another country on Iran's eastern border, is well known as a high *L. tropica* endemic country. The main foci are Kabul city and province, Herat, Badakhshan, and Kandahar with lesser burdens in Kunduz and Balkh provinces. The ACL rate in Kabul province is the highest in the country with a reported prevalence rate of 21% (108, 109). In Herat province, *L. tropica* accounts for 96 to 98% of CL patients (11, 110) and shares genetic characteristics with east and southeast Iranian strains (11, 111). Badakhshan and Kandahar provinces border Khyber Pakhtunkhwa and Baluchistan provinces of Pakistan, respectively, but there are no genetic comparative studies of *L. tropica*.

**Figure 6:**
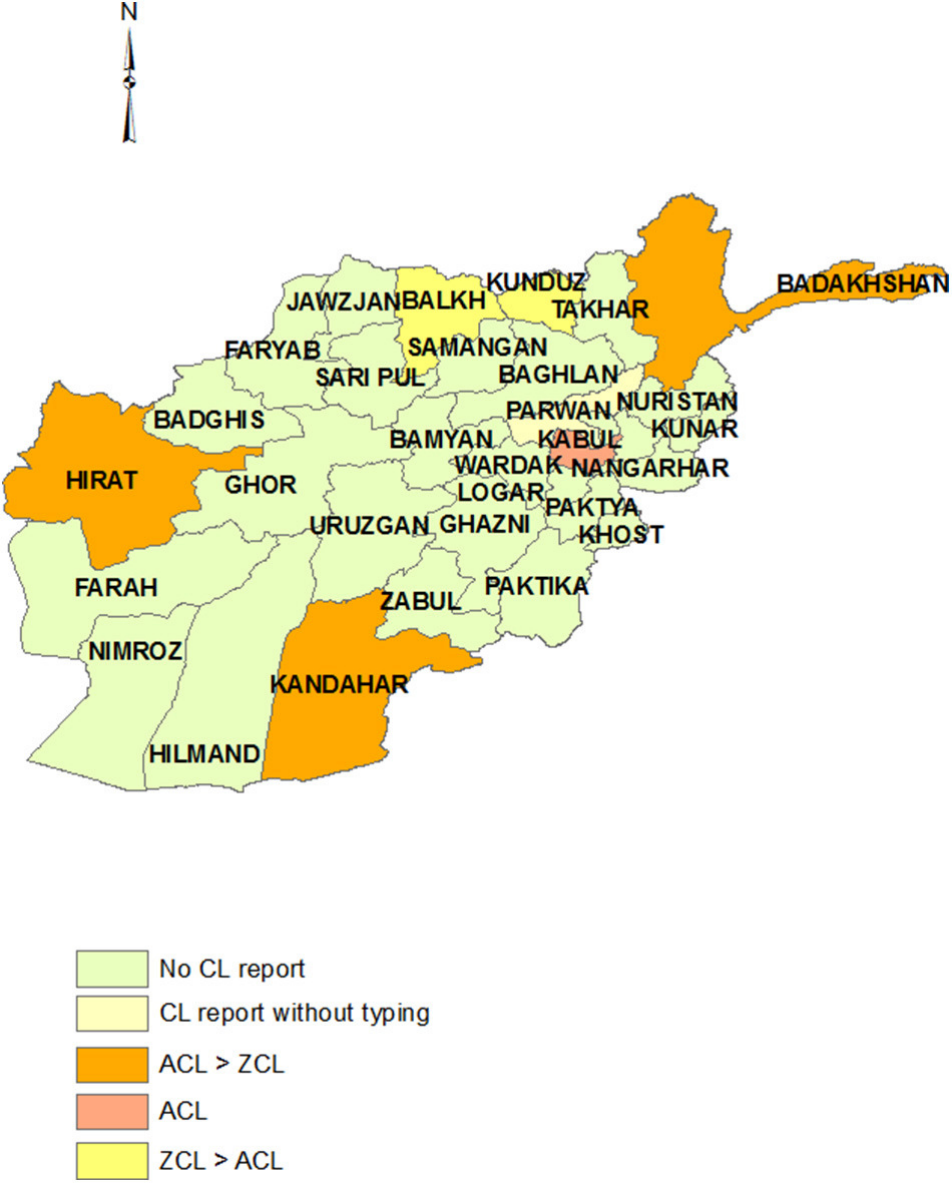
Distribution of CL types in Afghanistan.

*L. major* is reported as the minority species in Herat, Badakhshan, and Kandahar (112–114), but is more commonly found in Balkh and Kunduz provinces in northern Afghanistan along the border with Tajikistan (113). CL was reported from Parwan province in central Afghanistan but no data on the CL type has been published (Figure 6) (112). Afghanistan has suffered from political instability and periods of civil wars for decades, and a comprehensive view on the situation of CL in this country is not possible based on the existing data.

### Turkmenistan

Turkmenistan, Iran's northeastern neighbor, has been known historically as an ACL endemic country (115). The town of Ashkabad was an important focus but following concerted vector control efforts against the sandfly, the incidence of ACL fell but appears to be making a comeback (116, 117). Between 2000 and 2009, 1562 cases of CL were reported in Turkmenistan, mostly in the southern and eastern parts of the country bordering Iran and Afghanistan (118). *L. major* is also reported from time to time in the local population and travelers (117, 119).

### Syria

Recent years of conflict in Syria and the migration of large numbers of Syrian refugees to other countries have substantially affected the situation of leishmaniasis in the Middle East. By 1960, the prevalence of CL in Syria was limited to the two endemic regions of Aleppo (north) and Damascus (south) of Syria. In 2010, before the onset of the civil war, the incidence of CL was estimated at 23,000 cases a year (120), while after the onset of the war, the incidence of the disease almost doubled to 41,000 cases in 2013 (121). *L. tropica*-associated ACL is the main form of CL in Aleppo, whereas in Damascus, it is *L. major* ZCL (120). Overall, studies report that most cases of CL (about 90%) are caused by *L. tropica* (122). In 2009 (precivil war), of the 51,119 reported cases of CL in Syria, Jordan, Iraq, and Saudi Arabia, some 90.7% of cases were reported from Syria, making Syria the highest burden country for ACL in the region (123). Displacement of Syrian refugees to neighboring countries such as Lebanon, Iraq, Jordan, and Turkey has led to outbreaks of CL and altered the local epidemiology (124). In 2012, Saroufim et al. (125) sampled 948 Syrian patients residing in the refugee camps in Lebanon and reported that 85% of them were infected with *L. tropica* and 15% by *L. major*. According to the Lebanese Ministry of Health reports, only 6 cases of CL in Lebanon were reported between 2000 and 2012, while in 2011, 1033 new cases were reported; some 97% were Syrian refugees (124). Moreover, Syrian refugees have carried *L. major* and *L. donovani* to Turkey, two species that are not endemic to Turkey (83). Table 1 briefly shows the distribution of ACL and ZCL in the mentioned countries.

**Table 1:** Distribution of ACL and ZCL in different geographical parts of studied countries.

**Country**	**Region**	**ACL**	**ZCL**	**CL without species data**	**No CL data**
Iran	North			✓	
	Northeast	✓	✓		
	Northwest				✓
	Central	✓	✓		
	West	✓	✓		
	Southwest	✓	✓		
	South-southeast	✓	✓		
	East	✓	✓		
Iraq	North-Northeast				✓
	Northwest			✓	
	Central			✓	
	South-Southwest			✓	
	Southeast	✓	✓		
	East	✓	✓		
Turkey	North				✓
	Northeast				✓
	Northwest				✓
	South	✓	✓		
	Southwest-West	✓	✓		
	Southeast-East	✓	✓		
Saudi Arabia	North-Northwest				✓
	Northeast-East		✓		
	West (partly)			✓	
	South-Southwest	✓	✓		
	Central	✓	✓		
Afghanistan	West (partly)	✓	✓		
	South (partly)	✓	✓		
	North		✓		
	Northeast-East	✓	✓		
	Central				✓
Pakistan	South-Southeast-Southwest	✓	✓		
	West	✓	✓		
	North-Northwest	✓			
	East	✓			

## Discussion

**Figure 7:**
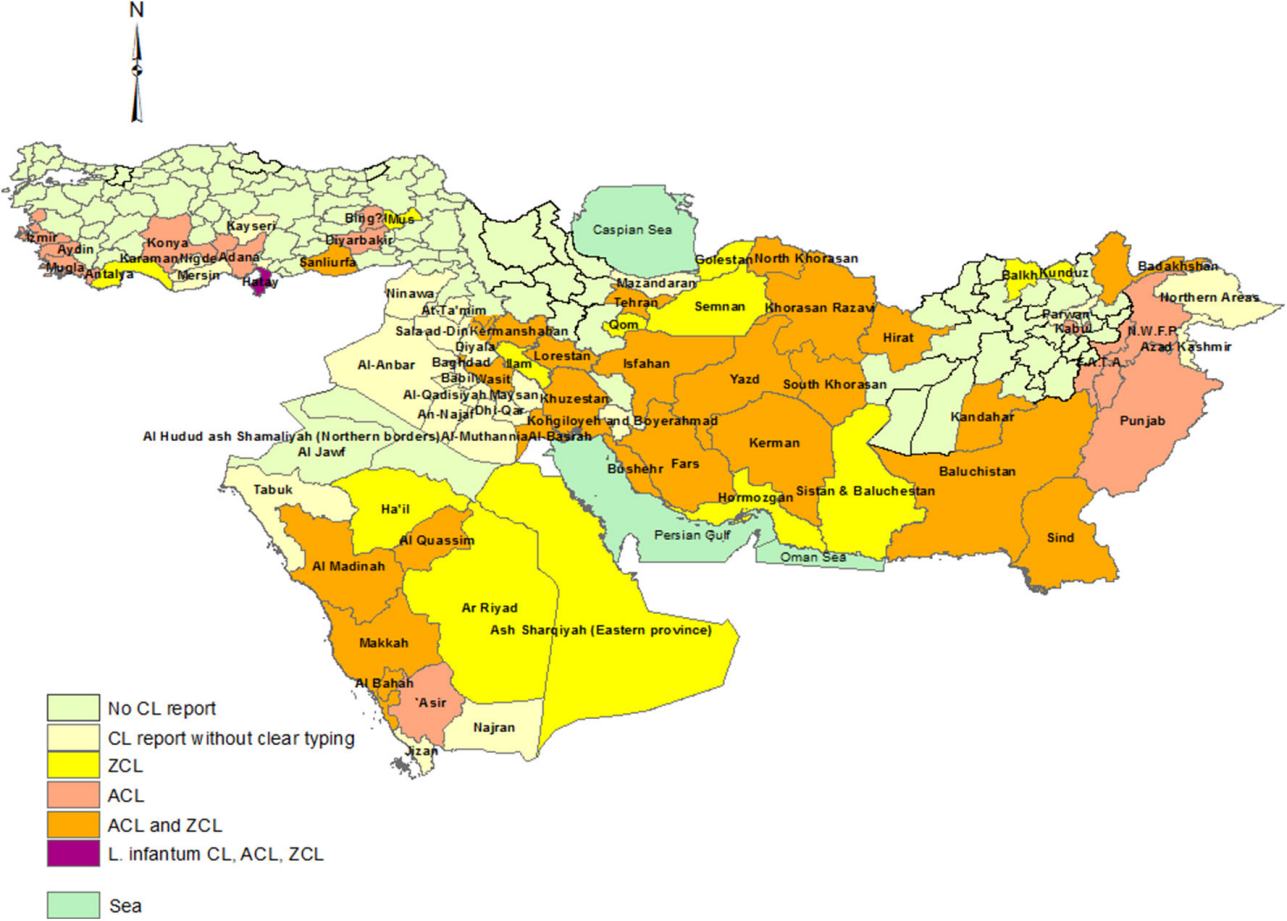
Distribution of CL types in the part of Middle East including Turkey, Iraq, Iran, Saudi Arabia, Afghanistan, and Pakistan in a view.

Our review reconfirms that CL remains an important disease in Iran and its neighboring countries (Figure 7). In this part of the Middle East, *L. major* ZCL and *L. tropica* ACL are present throughout the region in different ecological foci and with varying ratios of the two species. Data from 2012 suggest that Syria has the highest ACL burden followed by Iran, while Iran leads the ZCL burden followed closely by Saudi Arabia (126).

In this geographically heterogeneous region, CL has decreased in Iran and Saudi Arabia, increased dramatically in Syria, and re-emerged in Turkey and hitherto low endemic Lebanon. CL is geographically restricted to the southern coastline of Turkey and in selected provinces in Afghanistan while being extensively distributed in Iran, Saudi Arabia, and Iraq. Despite a reducing CL burden in Iran, new foci have merged including *L. tropica*, which is now reported from 10 provinces. Although *L. tropica* has long been known as an urban disease, it has expanded to rural areas and smaller towns in Iran (28, 127).

The relative lack of genotypic data makes it difficult to determine the patterns of spread and emergence in new foci. This is particularly important in border areas where traditionally there is much freedom of movement of populations. Phylogenetic data from Iran suggest that CL strains from SE Iran are related to strains from Herat in western Afghanistan, and strains from northwest Iran related to strains from SE Turkey (11, 28).

Moreover, the opening of the border between Iran and Iraq appears to have played an important role in the spread and the transmission of *Leishmania* strains between the two countries (128). High genetic similarity between a newly emerged Iraqi *L. major* strain and an Iranian strain support this hypothesis (101). As a vector-borne disease with two predominant vectors, *P. papatasi* (ZCL) and *P. sergenti* (ACL), and animal and human reservoirs, respectively, multiple factors will affect disease prevalence and epidemic proneness that will be favor one species over another or be common to both. Climate and ecology are particularly important.

Our investigations show that the risk of CL declines with increasing northern latitude, consistent with the findings of Alvar et al. (118) who reported the number of CL cases of 99, 17, and 0/year in Turkmenistan, Azerbaijan, and Armenia; all of these countries are north of Iran. Given the nature of the CL life cycle, Ready (129) suggested that climate change affects the distribution of leishmaniasis either directly by the effect of temperature on the development of the parasite in the sandfly, or indirectly by affecting the distribution and density of the vector species; added to these factors are the social and economic changes in human interaction with the transmission cycle. Long-term climate change has been shown to spread sandfly species to areas not previously observed (130), e.g., climate warming has caused sandflies to migrate to the high altitudes of the Atlas Mountains in Morocco (131).

The density of *P. papatasi*, a vector of *L. major*, increases with the aridity, which, in turn, occurs in response to global warming. Cross and Hyams have predicted that a 1 to 5°C increase in ambient temperature due to global warming will lead to the geographical spread of *P. papatasi* in the Middle East and an extension of the *L. major* transmission season to become perennial in Saudi Arabia (132). Currently, in Saudi Arabia, *P. papatasi* thrives best in the summer months and peak indoor activity occurs in May and peak density is seen in the late evening (10–11 p.m.); by winter, its population decreases drastically but starts to pick up in early spring in March (133). Martin-Sanchez et al. (134) have suggested that global warming has a greater effect on disease transmission rather than an increase in sandfly density. The effect of climate and ecology is illustrated well from studies in ZCL provinces of Iran where favorable conditions included mildly sloping hills of limited elevation, flat lands, irrigated farmlands, and moderate degrees of rainfall (41, 135). Consistent findings are reported by Ramezankhani et al. (135) who also report that lower wind speeds of <14 m/s were associated with increased disease incidence, independent of other climatic and geographical factors. In Golestan, a humid province in northeast Iran, ZCL prevalence was higher in areas with the lower relative humidity (136), whereas in Fars province, continued drought was associated with a decline in CL (137). Gerbils are important rodent reservoirs for supporting continued ZCL transmission and include *M. libycus* (the Libyan jird), *Psammomys obesus* (the fat tailed sand rat) in Saudi Arabia, *M. libycus, T. indica* (the Indian gerbil or antelope rat), and *R. opimus* (great gerbil) in Iran (133, 138). In a remarkable feat of evolution, *P. papatasi* has adapted to living in burrows inhabited by the fat tailed sand rat wherein high humidity and cooler temperatures are essential for sandfly survival; thus, this vector reservoir combination is a powerful driver of ZCL (139). Caves, deep cracks in walls, and dark corners in houses are also good for sandfly survival, even when with extreme temperature and aridity.

Population movements are another important risk for CL emergence, spread, and epidemics. These are related to non-immune populations moving into endemic areas or when CL-infected individuals move to a low endemic area with competent vectors; in both scenarios, population density is important. ZCL epidemics have been linked to construction workers moving into arid areas for building projects and American soldiers becoming infected in Iraq, while ACL epidemics have been reported in aid workers responding to the earthquake in Bam (SE Iran), large cities like Kabul, Afghan refugees in camps in Pakistan, and Syrian refugees in Lebanon and Turkey. Our region is well known for its religious pilgrimages and festivals, and each year, millions of pilgrims attend the Haj and Umrah in Makkah, the Arba'een pilgrimage in Karbala, Iraq, and Christmas in Jerusalem/Al Quds and Bethlehem in Israel and Palestine. This provides the opportunity for mixing of CL genotypes and non-immunes to become infected. War is a particularly effective way of causing CL epidemics not just because of population displacement but because the health infrastructure breaks down, resulting in very limited access to health care, destruction of buildings, and a deterioration in sanitation (140). The civil war in Syria is a striking example with large numbers from highly CL endemic foci causing epidemics in neighboring countries, as well as the emergence of new foci of CL caused by non-indigenous *Leishmania* species. Similarly, since 2014 in Iraq, population displacement has led to CL outbreaks in non-endemic areas between north and central Iraq (101). CL is found more commonly in poor and marginalized populations due to a variety of factors that include small overcrowded poorly constructed dwellings with no screens on windows, poor nutritional status, lack of personal protection against insect bites, and the presence of garbage collection areas (141).

Limited access to health and diagnosis due to lack of money or war-related infrastructure destruction and displacement exacerbate the situation.

Under-reporting of CL is probably widespread and data based on passive detection, as used in Iran (137), are significantly lower compared to active surveillance (142) because patients do not always seek treatment centers. In a study in Jordan, covering 2001–2003, the estimated incidence of CL was 47 times higher than official reports (142). Active surveillance for CL may be implemented only in emergencies such as outbreaks of the disease, like the case finding project of the Lebanese Ministry of Health, after the onset of the civil war in Syria and the influx of Syrian refugees to this country. Another issue is diagnosis. Some countries lack access to molecular techniques so species identification is impossible; thus, CL burden data are based on microscopy or the clinical description of lesions, e.g., dry or wet cutaneous lesions (109).

### Proposals to Reduce the Burden of the Disease in the Region

Surveillances should be improved by determining the species of *Leishmania* causing CL in each region by using molecular methods. Moreover, in the map reports, the distribution of the disease should be specified based on the species of the parasite. Using GIS and performing geospatial research are advised to stratify areas based on the probability of the disease. By using these data and producing hazard maps, the disease control and prevention can be better managed. Moreover, undertaking active disease screening in suspected areas of CL especially in refugee camps and increasing access to health care to remote areas and marginalized populations and introducing free treatment of CL can be the keyways to reducing the burden of CL in this region of the world.

## Conclusion

CL in the Middle East is a disease of complex interactions between parasite reservoirs, parasites, vectors, climate, ecology, political instability, poverty, and socioeconomic changes. Against this background is increasing and decreasing CL burden in different countries as well as newly emerging foci on low and high endemic countries. Effective control is challenging, must take all of these factors into account, and must adopt a multidisciplinary approach based on sound epidemiological evidence. This review sheds light on CL in our region with a focus on Iran, a country with a substantial burden.

## Author Contributions

MG conceptualized and wrote the initial draft. WT and MK critically appraised and revised the manuscript. All authors reviewed and approved the final manuscript.

### Conflict of Interest

The authors declare that the research was conducted in the absence of any commercial or financial relationships that could be construed as a potential conflict of interest.
